# Elevated Plasma Homocysteine Levels in Anti-N-methyl-D-aspartate Receptor Encephalitis

**DOI:** 10.3389/fneur.2019.00464

**Published:** 2019-05-03

**Authors:** Lizhi Liu, Jie Liang, Qing Liu, Chongliang Luo, Jia Liu, Rong Fan, Zhigang Chen, Yong Chen, Fuhua Peng, Ying Jiang

**Affiliations:** ^1^Department of Neurology and Multiple Sclerosis Research Center, The Third Affiliated Hospital, Sun Yat-sen University, Guangzhou, China; ^2^Department of Neurology, Huizhou Hospital of Traditional Chinese Medicine, Huizhou, China; ^3^Department of Biostatistics, Epidemiology and Informatics, University of Pennsylvania, Philadelphia, PA, United States

**Keywords:** anti-N-methyl-D-aspartate receptor encephalitis, homocysteine, modified Rankin Scale, C reactive protein, cerebrospinal fluid

## Abstract

**Objective:** Homocysteine (Hcy) levels have been investigated in many diseases, such as neurodegenerative and autoimmune diseases. However, changes in Hcy levels in anti-N-Methyl-D-aspartate receptor (anti-NMDAR) encephalitis have not been investigated thus far.

**Methods:** Case data were collected from 45 patients with anti-NMDAR encephalitis and 179 age- and sex-matched healthy controls (HCs). Clinical characteristics, Hcy levels, C reactive protein (CRP) levels, and cerebrospinal fluid (CSF) parameters were determined. Association of Hcy and clinical parameters were evaluated in these patients. Among these 45 patients, 15 had a follow-up evaluation at 3 months after treatment.

**Results:** Hcy levels (*p* < 0.001) and CRP levels (*p* = 0.005) from the patients with anti-NMDAR encephalitis were significantly higher than those from HCs. Hcy levels from male patients were significantly lower than those from male HCs (*p* < 0.001). Comparing anti-NMDAR encephalitis patients after treatment with before treatment, the former has significantly higher Hcy levels (*p* = 0.004), CRP levels (*p* = 0.041) and mRS scores (*p* = 0.002). Furthermore, a significant negative correlation between the changes in Hcy levels and the changes in mRS scores (*r* = −0.534, *p* = 0.040) was observed.

**Conclusion:** Elevated plasma homocysteine occurs in anti-NMDAR encephalitis, and seems associated with male sex.

## Introduction

Anti-N-methyl-D-aspartate receptor (anti-NMDAR) encephalitis is the most common antibody-mediated encephalitis (1), which commonly presents in young females of reproductive age and children, but also can occur in males and females of all ages with or without tumors, such as ovarian teratomas (2–4). This disorder, which represents severe neuropsychiatric manifestation characterized by seizures, memory decline, and behavioral deficits (5, 6), is caused by the production of autoantibodies against the GluN1 subunit of the NMDAR (7). Many studies have shown that immune cells, including B cells and T cells, play roles in the pathogenesis of anti-NMDAR encephalitis (8–11).

Homocysteine (Hcy) is a non-essential sulfur-containing amino acid and is considered a pro-inflammatory and immuno-modulating molecule (12). There is strong evidence that Hcy itself is able to induce the disruption of blood-brain barrier (BBB) (13) through NMDA receptor overstimulation and induce excitotoxicity on neurons (14–18) leading to caspase activation, DNA damage, and apoptosis (19).

Several studies have shown high Hcy levels occur in neurodegenerative disorders and autoimmune disorders, such as Alzheimer's and Parkinson's disease (20–22), multiple sclerosis (MS) (23–27), systemic lupus erythematosus (SLE) (28) and rheumatoid arthritis (RA) (29–31). However, the changes in Hcy levels in anti-NMDAR encephalitis were unclear. Therefore, we for the first time set out to investigate the association between Hcy levels and clinical parameters in anti-NMDAR encephalitis.

## Methods

### Study Design and Samples

This study is approved by the Medical Ethics Committee of the Third Affiliated Hospital of Sun Yat-sen University. The subjects or the guardians of patients with severe cognitive impairment provided written informed consent for research and publication. Lumbar puncture was also performed with informed consent.

We recruited 45 Chinese Han patients with anti-NMDAR encephalitis (male: female = 23:22) and 179 age- and sex-matched healthy controls (HCs) (male: female = 88:91) from January 2015 to December 2018. Diagnosis criteria for anti-NMDAR encephalitis were based on the diagnostic criteria by Graus et al. (32). Serum and cerebrospinal fluid (CSF) from patients were tested for IgG antibody against NMDAR by indirect immunostaining using a commercially available kit (EUROIMMUN Medizinische Labordiagnostika, Lübeck, Germany) according to the manufacturer's instructions. All patients were clinically evaluated for neurological status using the modified Rankin Scale (mRS) scores (33) and screened for systemic tumors with computed tomography (CT) or magnetic resonance imaging (MRI) or B ultrasound at least once. None of patients had obviously abnormal thyroid function, abnormal renal function or habit of smoking, excessive coffee or alcohol consumption. Additionally, these patients did not have excessive blood glucose at admission, thus they did not use hypoglycemic drugs. All the patients involved in this study were diagnosed as anti-NMDAR encephalitis for the first time and they did not receive treatments such as vitamin B12, folic acid, vitamin B6, glucocorticoids, methotrexate and methionine before admission. Only 6 of the 35 patients with seizure were treated with antiepileptic drugs (AEDs) for a short term (<1 week) before admission. In these six patients, two were treated with valproic acid (VPA), two with lamotrigine (LTG), one with carbamazepine (CBZ) and one with levetiracetam (LEV).

### Laboratory and Clinical Assessment

Venous blood was collected from patients with anti-NMDAR encephalitis on the next day at 7:00 to 8:00 a.m. after their admission. Plasma Hcy levels were determined by high-performance liquid chromatography with fluorescence detection. Hyperhomocysteinaemia has been defined as a Hcy concentration ≥10 μmol/L. The cutoff Hcy level of < 10 μmol is recommended by the American Heart Association and the American Stroke Association Council on Stroke (34, 35). We also examined C reactive protein (CRP) and CSF parameters, including white blood cells (WBC), total protein (TP), glucose (GLU) and chloride (CL). Brain MRI was also reviewed.

### Follow-Up Evaluations

Among these 45 anti-NMDAR encephalitis patients, 15 who received treatments including tumor removal, the first-line immunotherapy such as steroids, IVIG, alone or combined, and the second-line immunotherapy such as rituximab, azathioprine, and cyclophosphamide, alone or combined. These 15 patients had a follow-up evaluation 3 months after admission. The follow-up evaluation included the repetition of mRS scores and the measurement of CRP and Hcy levels.

### Statistical Analyses

All statistical analyses were performed using the Statistical Program for Social Sciences (SPSS) software (version 22.0, Chicago, IL, USA). The data in this study were presented as mean ± standard deviation (SD) if the data was normally distributed or as median and interquartile range (IQR) if the data was not normally distributed. Unless otherwise noted, we used student *t*-test for testing the difference of normally distributed variable from two groups, Mann-Whitney U test (also known as Wilcoxon rank-sum test) for testing the difference of non-normally distributed variable from two groups, and Chi-square test for testing the association of two binary variables. Paired *t*-test was used for normal data and paired Mann-Whitney U test was used for non-normal data. All tests were two-sided with a significant level of 0.05.

## Results

### Clinical Characteristics of Anti-NMDAR Encephalitis Patients and Healthy Controls

Table 1 shows anti-NMDAR encephalitis patients (female: male = 23:22) were comparable to HCs (female: male = 88:91), with median age of 30.64 years compared to 30.96 years in HCs. The median disease duration and mRS score in the anti-NMDAR encephalitis patients were 28.29 ± 14.33 days and 4 (3.00–4.00), respectively. Nine of 45 patients (20%) had tumors. Thirty five of 45 patients (77.8%) had seizure.

**Table 1:** Demographic features of patients with anti-NMDAR encephalitis and healthy controls.

	**Anti-NMDAR encephalitis (*n* = 45)**	**Healthy controls (*n* = 179)**	P^*^ value
Age onset (y, mean ±*SD*)	30.64 ± 15.94	30.96 ± 14.00	0.895^P1^
Gender (male: female)	23:22	88:91	0.815^P3^
Disease duration (d, mean ±*SD*)	28.29 ± 14.33	–	
CSF anti-NMDAR Abs positive (*n*, %)	45(100)	–	
**HCY level (μmol/L, IQR)**		
Total	9.64 (7.49–12.31)	8.32 (6.79–9.44)	<0.001^P1^
**Gender**		
Male	11.80 (9.49–16.05)^a^	8.88 (7.40–9.67)^b^	<0.001^P1^
Female	8.33 (6.59–10.90)^c^	7.67 (6.08–9.24)^d^	0.173^P1^
**Age (IQR)**		
<18 y	10.73 (7.66–12.38)^e^	8.33 (7.09–9.77)^f^	0.199^P2^
≥18 y	9.64 (7.32–13.91)^g^	8.32 (6.65–9.41)^h^	0.001^P1^
CRP level (mg/L, IQR)	1.9 (0.30–7.66)^i^	0.8 (0.40–1.83)^j^	0.005^P2^
mRS (IQR)	4 (3–4)	–	
With seizure (*n*, %)	35 (77.8)	–	
With tumor (*n*, %)	9 (20)	–	

Anti-NMDAR, anti-N-Methyl-D-aspartate receptor; CSF, cerebrospinal fluid; Ab, antibody; Note: Hcy, homocysteine; mRS, modified Rankin Scale; SD, standard deviation, IQR, interquartile range. a, n = 23; b, n = 88; c, n = 22; d, n = 91; e, n = 10; f, n = 20; g, n = 35; h, n = 159; i, n = 43; j, n = 136.

* All continuous variables were presented as the mean (± standard deviation) if the data was normally distributed or as median values and quartiles if the data was not normally distributed. To assess the significance of differences between groups, the Student's t test was applied when the data were normally distributed, while Mann-Whitney U tests were performed when the data were not normally distributed. Chi-square tests were used for testing the association of two binary variables. P^*^ value: P1, the Student's t-test; P2, Mann-Whitney U tests; P3, Chi-square test.

### Comparison Between Anti-NMDAR Encephalitis Patients and Healthy Controls

**Figure 1:**
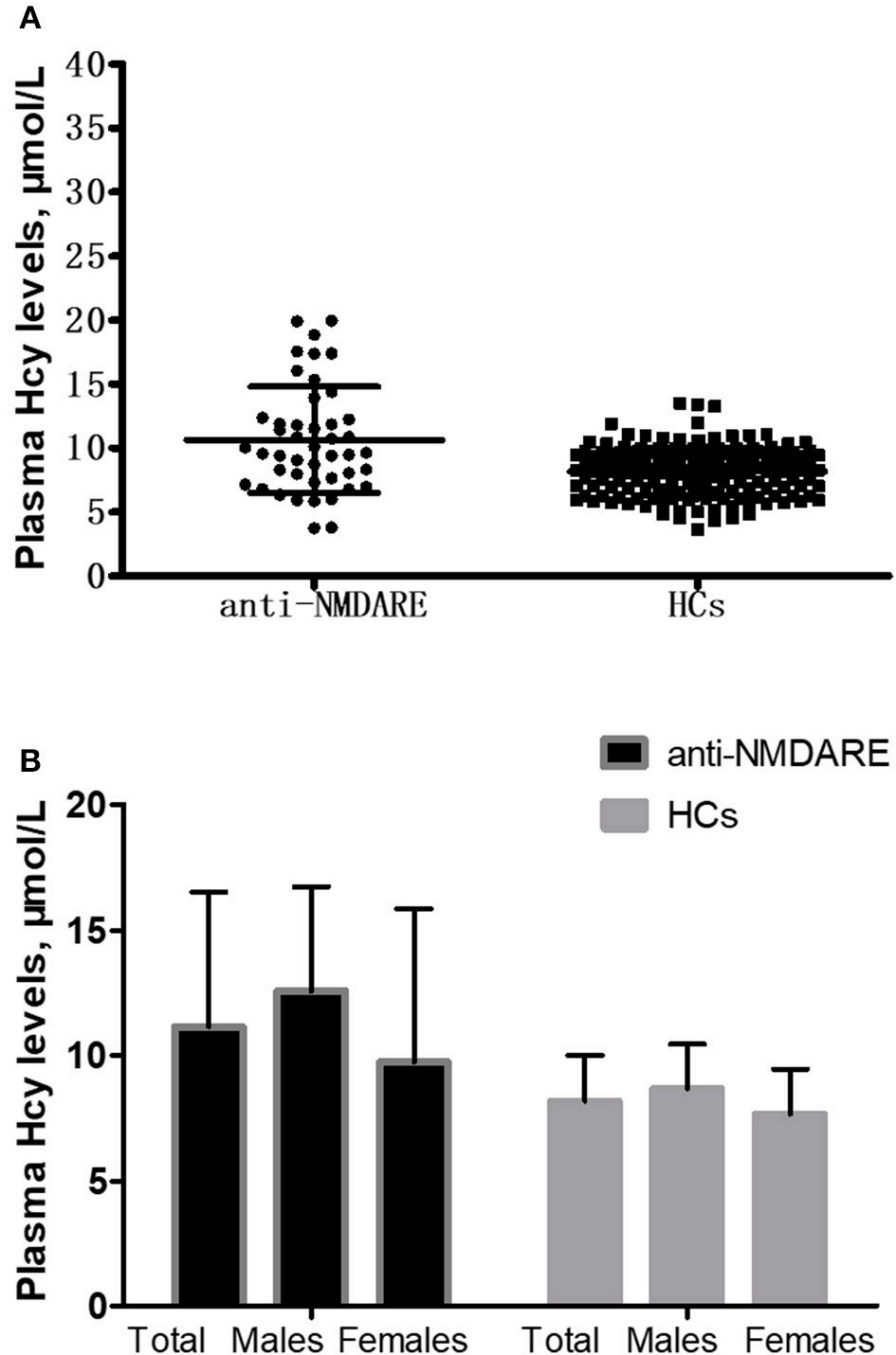
Comparison of homocysteine (Hcy) levels between anti-NMDAR encephalitis (anti-NMDARE) patients and healthy controls (HCs). **(A)** Comparison of Hcy levels between the anti-NMDAR encephalitis patients (*n* = 45) and healthy controls (HCs) (*n* = 179), *p* < 0.001. **(B)** Comparison of Hcy levels between the anti-NMDAR encephalitis patients and HCs according to gender. Anti-NMDAR encephalitis patients (male) vs. HCs (male), *p* < 0.001; anti-NMDAR encephalitis patients (female) vs. HCs (female), *p* = 0.173.

The mean Hcy levels of anti-NMDAR encephalitis patients were significantly higher than the mean Hcy level of HCs (*p* < 0.001; Table 1 and Figure 1A). In addition, the mean CRP levels of anti-NMDAR encephalitis patients were also statistically significantly higher than HCs (*p* = 0.005; Table 1). Hcy levels were significantly higher in male patients than in male HCs (*p* < 0.001; Table 1 and Figure 1B) and higher in patients with age ≥18 years than in HCs with age ≥18 years (*p* = 0.001; Table 1).

### Comparisons Between Different Subgroups of Anti-NMDAR Encephalitis Patients

Patients with anti-NMDAR encephalitis were divided into different subgroups according to gender, age, mRS, disease duration, brain MRI, presence of tumor and presence of seizure, and then we investigated Hcy levels in different subgroups. As summarized in Table 2, male patients had significantly higher Hcy levels than female patients (*p* = 0.001), and patients with seizure had significantly higher Hcy levels than patients without seizure (*p* = 0.005). Though there were no differences in Hcy levels in patients with and without tumor, when we further divided these patients without tumors into male and female subgroups, we found that Hcy levels in the male group is significantly higher than the female group (12.49 ± 4.03 μmol/l vs. 8.83 ± 3.19 μmol/l, *p* = 0.005).

**Table 2:** Plasma Hcy levels in patients with anti–NMDAR encephalitis.

**Variables**	**Value (μmol/L)**	**Range (min–max, μmol/L)**	P^*^ value
**GENDER**			
Male (IQR, *n =* 23)	11.80 (9.49–16.05)	6.36–19.97	
Female (IQR, *n =* 22)	8.33 (6.59–10.90)	3.74–17.41	0.001^P2^
**AGE**			
<18 y (IQR, *n =* 10)	9.90 (7.49–11.65)	3.80–15.35	
≥18 y (IQR, *n =* 35)	9.64 (7.32–13.91)	3.74–19.97	0.401^P2^
**mRS**			
<4 (IQR, *n =* 16)	10.45 (7.82–13.53)	3.80–17.37	
≥4 (IQR, *n =* 29)	9.49 (7.24–12.06)	3.74–19.97	0.975^P2^
**DISEASE DURATION (d)**			
>30 (mean ±*SD, n =* 19)	11.11 ± 5.26	3.74–19.97	
≤30 (mean ±*SD, n =* 26)	10.30 ± 3.18	5.93–18.86	0.557^P1^
**BRAIN MRI**			
Normal (mean ±*SD, n =* 25)	10.12 ± 5.02	3.74–17.55	
Abnormal (mean ±*SD, n =* 20)	11.30 ± 5.02	3.80–19.97	0.373^P1^
**TUMOR**			
With (mean ±*SD, n =* 9)	9.76 ± 4.61	3.74–19.97	
Without (mean ±*SD, n =* 36)	10.87 ± 4.07	3.80–19.9	0.482^P1^
**SEIZURE**			
With (mean ±*SD, n =* 35)	11.32 ± 4.36	3.74–19.97	
Without (mean ±*SD, n =* 10)	8.29 ± 2.15	3.80–11.55	0.005^P1^

*Anti-NMDAR, anti-N-Methyl-D-aspartate receptor; Hcy, homocysteine; mRS, modified Rankin Scale; SD, standard deviation, IQR, interquartile range*.

* All continuous variables were presented as the mean ( ± standard deviation) if the data was normally distributed or as median values and quartiles if the data was not normally distributed. To assess the significance of differences between groups, the Student's t test was applied when the data were normally distributed, while Mann–Whitney U tests were performed when the data were not normally distributed. P^*^ value: P1, the Student's t-test; P2, Mann–Whitney U tests.

### Comparison Between Hcy Concentration ≥10 μmol/L and Hcy Concentration < 10 μmol in Anti-NMDAR Encephalitis Patients

In this study, we defined 10 μmol/L as cutoff points for hyperhomocysteinaemia (Hcy ≥10 μmol/L and Hcy < 10 μmol/L). Except for gender (*p* = 0.005), there were not any statistically significant differences in disease duration, mRS scores, CRP, CSF parameters between Hcy ≥10 μmol/L and Hcy < 10 μmol/L and seizure in anti-NMDAR encephalitis patients (Table 3).

**Table 3:** Comparison between Hcy concentration ≥ 10 μmol/L and Hcy concentration < 10 μmol in anti-NMDAR encephalitis patients.

	**Anti-NMDAR encephalitis (*****n*** **=** **45)**		
	**Hcy level (μmol/L)≥10**	**Hcy level (μmol/L) <10**	P^*^ value
CSF anti-NMDAR Abs positive (*n*, %)	22 (48.99)	23 (51.11)	–
Gender (male: female)	16:6	7:16	0.005^P3^
Disease duration (d, mean ±*SD*)	28.18 ± 14.41	28.39 ± 14.58	0.962^P1^
mRS (IQR)	4 (3–4)	4 (3–5)	0.422^P2^
CRP (mg/L, IQR)^a^	1.40 (0.30–5.15)	2.8 (0.35–13.30)	0.243^P2^
**CSF PARAMETERS**			
CSF WBC ( × 10^6^/L, IQR)^a^	6.0 (2.3–49.0)	14.0 (6.0–29.0)	0.323^P2^
CSF TP (g/L, IQR)^a^	0.315 (0.20–0.54)	0.26 (0.17–0.41)	0.381^P2^
CSF GLU (mmol/L, mean ±*SD*)^b^	3.66 ± 0.76	3.53 ± 0.91	0.608^P1^
CSF CL (mmol/L, mean ±*SD*)^b^	122.76 ± 3.88	121.39 ± 4.13	0.278^P1^
With seizure (*n*, %)	20 (90.9)	15 (65.2)	0.087^P3^

*Anti-NMDAR, anti-N-Methyl-D-aspartate receptor; CSF, cerebrospinal fluid; Ab, antibody; mRS, modified Rankin Scale; WBC, white blood cells; TP, total protein; GLU, glucose; CL, chloride; CRP, C-reactive protein. a, n = 43; b, n = 42*.

* All continuous variables were presented as the mean (± standard deviation) if the data was normally distributed or as median values and quartiles if the data was not normally distributed. To assess the significance of differences between groups, the Student's t test was applied when the data were normally distributed, while Mann-Whitney U tests were performed when the data were not normally distributed. Chi-square tests were used for testing the association of two binary variables. P^*^ value: P1, the Student's t test; P2, Mann-Whitney U tests; P3, Chi-square test.

### Correlation Between Hcy Level and Clinical Parameters in Anti-NMDAR Encephalitis Patients

In the analysis of the associations between Hcy level and clinical characteristics, as well as CRP and CSF parameters, we did not find any significant correlations between Hcy level and these variables. We further divided anti-NMDAR encephalitis patients into two groups (females/males). However, we also did not find any significant correlations between Hcy level and these variables.

### Follow-Up Evaluation of Hcy Level in Anti-NMDAR Encephalitis Patients Following Treatments

**Figure 2:**
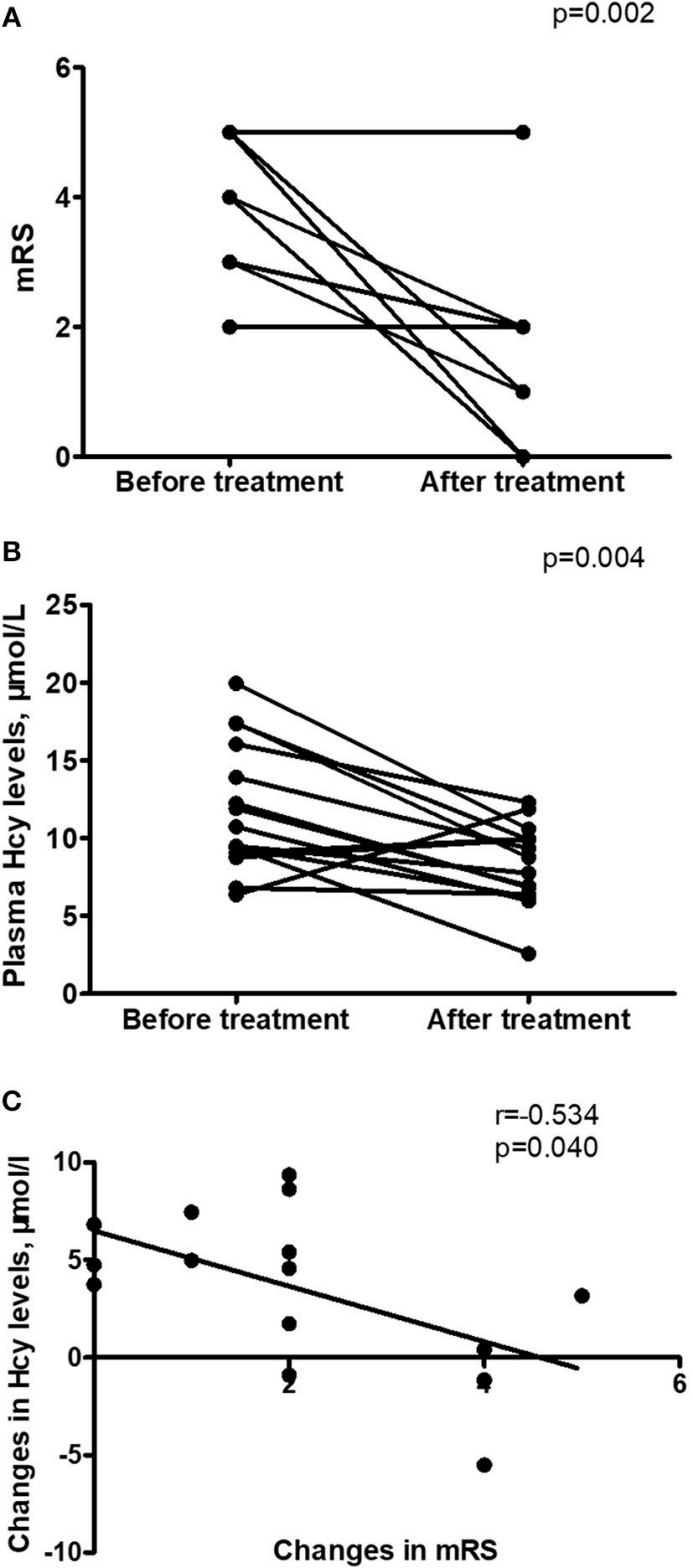
Changes in homocysteine (Hcy) levels in anti-NMDAR encephalitis patients after treatment. **(A)** Improvement in modified Rankin Scale (mRS) scores from initial admission to 3-month follow-up after treatment. **(B)** Changes in Hcy levels from initial admission to 3-month follow-up after treatment. **(C)** Relationship between the changes in mRS scores and the changes in Hcy levels after treatment (*r* = −0.534, *p* = 0.040). *n* = 15.

Hcy levels, CRP levels and mRS scores were significantly lower in follow-up evaluation 3 months after treatment than in initial admission, as shown in Table 4 and Figures 2A,B. Furthermore, the reduction of Hcy levels had a significantly negative correlation with the reduction of mRS scores after treatment (*r* = −0.534, *p* = 0.040; Figure 2C).

**Table 4:** Demographics of the 15 anti-NMDAR encephalitis with 3-month follow-up.

	**Anti-NMDAR encephalitis (*****n*** **=** **15)**		
	**Before treatment**	**After treatment**	P^*^ value
CSF anti-NMDAR Abs positive (n, %)	15 (100)	12 (80)	0.224^P1^
Sex (male: female)	8:7	8:7	–
mRS (IQR)	4 (3–5)	2 (1–2)	0.002^P2^
Hcy level (umol/L, IQR)	10.73 (6.36–19.97)	8.77 (2.57–12.30)	0.004^P3^
CRP level (mg/L, IQR)^a^	7 (0.65–13.45)	1 (0.60–4.90)	0.041^P2^

*Anti-NMDAR, anti-N-Methyl-D-aspartate receptor; CSF, cerebrospinal fluid; Ab, antibody; Hcy, homocysteine; mRS, modified Rankin Scale; CRP, C-reactive protein; IQR, interquartile range. a, n = 13*.

* Chi-square test was used for testing the association of two binary variables. Paired t-test was used when comparing the Hcy levels before and after treatment, and paired Mann-Whitney U test (also known as Wilcoxon rank-sum test) was used when comparing mRS scores and CRP level before and after treatment. P^*^ value: P1, Chi-square test; P2, paired Mann-Whitney U tests; P3, paired t-test.

## Discussion

To the best of our knowledge our study is the first to analyze Hcy levels in anti-NMDAR encephalitis patients. In this study, we found that significantly higher Hcy levels were associated with anti-NMDAR encephalitis patients, male gender and seizure in patients. In the follow-up evaluation, 15 anti-NMDAR encephalitis patients had decreased Hcy levels and mRS scores after treatment. Furthermore, the reduction of Hcy levels had a significantly negative correlation with the reduction of mRS scores after treatment.

Anti-NMDAR encephalitis is an immune-mediated inflammatory disorder. Immune cells, including B cells and T cells, play roles in the pathogenesis of anti-NMDAR encephalitis (8–11). Increased Hcy levels were observed in the overall sample of our cases with anti-NMDAR encephalitis when compared to those in HCs. This result is similar to the findings in other autoimmune disorders, such as MS (23–27), SLE (28), and RA (29–31). We also found increased Hcy level accompanying the increased CRP level, which is an immune inflammatory marker. There are similar reports about higher levels of Hcy and CRP in MS and SLE patients with metabolic syndrome (27, 28). Additionally, we also found a significant 18% Hcy reduction in follow-up evaluation 3 months after treatment. This finding together with the concomitant decrease in CRP level in this study provided a further indirect evidence of the link between inflammation and high Hcy level in anti-NMDAR encephalitis patients, which is similar to the findings in RA patients (29). Hcy is able to stimulate CRP production and then initiate an inflammatory response in vascular smooth muscle cells (VSMCs) (36). A decrease in Hcy level after treatment would be expected to accompany with the overall reduction in inflammation status. The specific mechanism behind the association between high Hcy level and anti-NMDAR encephalitis is unclear. However, lots of studies have showed that Hcy in excess can cause neurotoxicity by its dual actions on NMDAR (37) and Hcy may also induce excitotoxicity on neurons through overstimulation of NMDAR (14–18). We suppose that high Hcy levels may cause an imbalance in the immune system, less protection against inflammatory response and neurotoxicity in anti-NMDAR encephalitis.

Age and gender are two main determinants of Hcy concentrations in humans (38). In adults, plasma concentration of Hcy generally increases with age (38). However, we didn't find statistically significant difference in Hcy levels between < 18 years and ≥18 years in the entire cohort of patients, which might be due to small sample size, especially the number of the patients under the age of 18 years (*n* = 10). In line with several studies on MS, schizophrenia and Behcet's disease (26, 39–41), this study also showed that Hcy level is significantly higher in male patients with anti-NMDAR encephalitis but not in female patients. Messedi et al. (41) confirmed that 677 T allele and male gender were independent risk factors for hyperhomocysteinemia in Behcet's disease patients. This finding might involve the synthesis of Hcy along with creatine/creatine synthase, which is proportional to the difference in muscle mass that is higher in men than in women (42). Furthermore, higher estrogen level was associated with the decreased of total Hcy concentration in serum, independent of nutritional status and muscle mass. Therefore, variations of estrogen levels might explain the difference of total Hcy concentration among males and females (43). Additionally, the MTHFR 677C>T polymorphism appears to have a sex-specific effect on homocysteine (44). Men who are MTHFR 677TT homozygotes appear to be at higher risk of hyperhomocysteinemia than women with this genotype (45). However, MTHFR 677C>T genotype have not been examined in this research. It is one of the limitations of this study and needs further research. In fact, the mechanisms about the role of Hcy in NMDAR encephalitis in patients without tumor are unknown. A bi-directional link seems to connect Hcy and the immuno-inflammatory activation characterizing autoimmune diseases, in which immuno-inflammatory activation may contribute to Hcy increase, and Hcy, in its turn, may act as a pro-inflammatory and immuno-stimulating molecule putatively cooperating at the injury of the disease-specific target organs. Moreover, Hcy may be also a trigger of autoimmune reactions through its capability to bind and structurally modify specific proteins, then resulting in neoantigens formation, potentially relevant in the onset of specific autoimmune diseases (12).

It's well-known that AEDs may affect the metabolism of Hcy levels. Studies showed long-term treatment with some AEDs, including VPA and CBZ, may lead to hyperhomocysteinemia (46, 47). In contrast, LTG and LEV were found neutral with regard to Hcy concentrations (48). However, there is limited data about the effect of a short-term period of AEDs treatment on Hcy levels. One study showed no statistically significant changes were seen in Hcy levels before VPA therapy and after 1 week, 1 month of VPA treatment (49). In general, the samples were obtained during steady-state conditions, i.e., after more than 1 month of therapy with AEDs. Thus, the elevated levels of Hcy in these anti-NMDAR encephalitis patients with seizure should not been influenced by a short-term period of AEDs treatment (< 1 week). Actually, NMDAR plays an important role in the generation and maintenance of epileptic seizures. Hcy can trigger epileptic fits as agonists for NMDA receptors (50) and the administration of high doses of Hcy in animal models can produce convulsive seizures (51–53). Therefore, elevated Hcy level probably may reduce the seizure threshold in some susceptible patients, such as anti-NMDAR encephalitis.

Our study has several limitations. Firstly, the numbers of patients were relatively small and only a single ethnic population from a single center was evaluated. Secondly, lifestyle conditions such as physical inactivity are not collected in this study, which may play a role in modulating Hcy level, although the evidence remains controversial. Another limitation is that we did not analyze MTHFR 677C>T genotype, folate or vitamin B12 in our study, which are possible determinants of homocysteine levels.

In this study, we found that results of Hcy level were significantly different between anti-NMDAR encephalitis patients and HCs. Elevated plasma homocysteine occurs in anti-NMDAR encephalitis, and seems associated with male sex.

## Ethics Statement

The study was conducted according to the principles expressed in the Declaration of Helsinki and approved by the Medical Ethics Committee of the Third Affiliated Hospital of Sun Yat-sen University. All study participants gave written informed consent for research and publication.

## Author Contributions

YJ and FP contributed to the conception and design of this study. LL, JLiang, YJ, QL, JLiu, RF, ZC, and FP collected and organized the data. YJ, LL, JLiang, CL, YC, RF, and QL analyzed the data. YJ, LL, JLiang, QL, CL, YC, and FP drafted the manuscript. All the authors read and approved the final manuscript.

### Conflict of Interest Statement

The authors declare that the research was conducted in the absence of any commercial or financial relationships that could be construed as a potential conflict of interest.
